# 

**Published:** 1889-03

**Authors:** 


### TABLE OF CONTENTS.

ORIGINAL AND SELECTED.

Cases in Practice, T. L. Lallerstedt, M. D....... 81
Diphtheria &c., J. W. Duncan, M. D............ 82
Metrorrhagia, P. R. Cortelyou M. D. .......... 84
A law of Heridity, &c...... ............... 87
Dr. T. A. Cravens reports, &c. ...... .......... 89
The use of Forceps, &c....... ............... 91
Antisepsis and the Country Physician......... 92
A note on the Treatment of Migraine ...... 93
Salol in the Treatment of Cystitis, E. L. Vansant, M. D. ........................... 95

ABSTRACTS &c.

Sulfanol, the new Hypnotic ........... 96
The Arsenite of Copper in Cholera Morbus; Strychnia an antidote for Morphine Poisoning .................. 97
The action of Chloroform water, &c. ......... 98
On some Peculiar Effects of Circumcision ..... 99
Hydriotic Acid; The use of Ethylate of sodium, in the Treatment of Lupus ............ 100
Night Terror, &c. ................ 101
Digitalis in Remittent Fever; Pichi in Cystitis 102
Treatment of Sprains; Chronic Ulcer of the Leg ................................ 103
Climatic Influence on the Morals ........ 104
Lanolin &c.; Treatment of Incontinence of Urine ................................ 105
Inflammatory Sore Eyes.................... 106
Catarrhal Conjunctivitis; Purulent Conjunctivitis ............................ 107
Amputation of the Pregnant Uterus........... 108
A new Antiseptic Surgical Dressing ........ 109
Disinfection of Surgeons' Hands; Epilepsy; Iodine for Worms ................ 110

SCIENTIFIC

Whence the Corona?; Glass Cloth ........ 111
Chinese have no Nerves; The sound of Thunder; The Total Solar Eclipse of the Sun, Jan. 1 ............................ 112

NOTES AND FORMULÆ.

Ointment for Hemorrhoids; Seborrhœa; Menorrhagia.......................... 113
Amenorrhœa; Ringworm; Epilation, Removal of Crusts, etc.; To prevent Scarlet Fever; A Hair Restorer; For the Constipation of Children.... .......................... 114

EDITORIAL.

Southern Medical College Commencement; Cineraria Maritima; Iodoform Bituminate; Tribromphenol ........................ 115
Decline in Quinine ...................... 116
State Laws for the Regulation of the practice of Medicine; Death of Wm. Weightman: Excessive Medication ............ 117
The Atlanta Society of Medicine; Insanity Amongst the Negroes ................ 118
Advertisers, &c.................. ...... 116, 117, 118
Special Notes ......................... 119, 120

